# Viral Infection and Cardiovascular Disease: Implications for the Molecular Basis of COVID-19 Pathogenesis

**DOI:** 10.3390/ijms22041659

**Published:** 2021-02-07

**Authors:** Sarah Seeherman, Yuichiro J. Suzuki

**Affiliations:** 1 College of Osteopathic Medicine, Lake Erie College of Osteopathic Medicine, Erie, PA 16509, USA; SSeeherman77608@med.lecom.edu; 2 Department of Pharmacology and Physiology, Georgetown University Medical Center, Washington, DC 20007, USA

**Keywords:** ACE2, cardiovascular, coronavirus, COVID-19, dengue, heart, HIV, influenza, spike protein, virus

## Abstract

The current pandemic of coronavirus disease 2019 (COVID-19) is caused by severe acute respiratory syndrome coronavirus 2 (SARS-CoV-2). While this respiratory virus only causes mild symptoms in younger healthy individuals, elderly people and those with cardiovascular diseases such as systemic hypertension are susceptible to developing severe conditions that can be fatal. SARS-CoV-2 infection is also associated with an increased incidence of cardiovascular diseases such as myocardial injury, acute coronary syndrome, and thromboembolism. Understanding the mechanisms of the effects of this virus on the cardiovascular system should thus help develop therapeutic strategies to reduce the mortality and morbidity associated with SARS-CoV-2 infection. Since this virus causes severe and fatal conditions in older individuals with cardiovascular comorbidities, effective therapies targeting specific populations will likely contribute to ending this pandemic. In this review article, the effects of various viruses—including other coronaviruses, influenza, dengue, and human immunodeficiency virus—on the cardiovascular system are described to help provide molecular mechanisms of pathologies associated with SARS-CoV-2 infection and COVID-19. The goal is to provide mechanistic information from the biology of other viral infections in relation to cardiovascular pathologies for the purpose of developing improved vaccines and therapeutic agents effective in preventing and/or treating the acute and long-term consequences of SARS-CoV-2 and COVID-19.

## 1. Introduction

Severe acute respiratory syndrome coronavirus 2 (SARS-CoV-2) is causing the current pandemic of coronavirus disease 2019 (COVID-19). SARS-CoV-2 had infected at least 100 million people and taken over two million lives globally as of January 2021. To help understand the pathophysiology of COVID-19 that involves cardiovascular complications, this review article gathers information on the effects of various viruses on the cardiovascular system and diseases with a focus on molecular mechanisms to raise scholarly understanding of COVID-19 and help find therapeutic strategies to end this pandemic.

## 2. SARS-CoV-2: The Global Problem

SARS-CoV-2 is a single-stranded, positive-sense RNA virus [1,2]. It uses its spike protein to attach to host cells using the angiotensin-converting enzyme 2 (ACE2) as its receptor for membrane fusion [3,4]. The spike protein contains two functional subunits. The S1 subunit contains the receptor-binding domain that binds to ACE2 and the S2 subunit is responsible for the fusion and entry into the host cell. Before entry, the spike protein must be primed by a serine protease TMPRSS2 [5].

ACE2, part of the renin-angiotensin system, is responsible for cleaving angiotensin (Ang) II into Ang (1-7) [6,7]. Ang II binding to Ang receptors causes vasoconstriction, inflammation, vascular remodeling and fibrosis [8]. Ang (1-7) binds to the MAS receptor causing vasodilation [9]. Once SARS-CoV-2 binds to ACE2 via the spike protein, the genetic materials of SARS-CoV-2 enter the host cell where replication and amplification occur [3]. This is the same mechanism as that used by severe acute respiratory syndrome coronavirus (SARS-CoV; now also known as SARS-CoV-1) [10]. ACE2 is abundantly expressed in type 2 alveolar epithelial cells, which explains the lung complications seen with SARS-CoV-2. It is also highly expressed in the digestive tract, gallbladder, kidneys, brain, and vasculature—including the arterial and venous endothelial cells and arterial smooth muscle cells [11]. The involvement of ACE2 likely contributes to this respiratory virus exhibiting cardiovascular complications.

The involvement of ACE2 in cardiovascular diseases has been extensively studied, as it regulates the level of Ang II, a potent vasoconstrictor, a mediator of cardiac and vascular remodeling and an inducer of fibrosis [12]. ACE2-deficient mice have been shown to exhibit severe reduction in cardiac contractility [13], increased blood pressure, and an enhanced susceptibility to Ang II-induced hypertension [14]. On the contrary, the overexpression of ACE2 attenuates Ang II-induced hypertension [15] and protects the heart from hypertension-induced cardiac remodeling by inhibiting both myocardial and perivascular fibrosis [16]. It has been shown that ACE2 activity increases when hypertension develops and further increases when the disease progresses to systolic dysfunction [17]. Moreover, ACE2 is overexpressed in the paraventricular nucleus in spontaneously hypertensive rats while ACE2 gene transfer results in a significant attenuation of high blood pressure. ACE2-treated spontaneously hypertensive rats show a significant reduction in left ventricular wall thickness and perivascular fibrosis [18].

While this respiratory virus largely causes mild symptoms in younger healthy individuals, elderly people and those with cardiovascular comorbidities such as systemic hypertension are susceptible to developing severe and possibly fatal COVID-19. Infection with SARS-CoV-2 is also associated with an increased incidence of the development of cardiovascular diseases such as myocardial injury, acute coronary syndrome, and thromboembolism. The topic of COVID-19 and cardiovascular disease has been reviewed by others [19,20,21]. In this article, information from other viruses is analyzed to help understand molecular basis of cardiovascular pathologies in COVID-19.

## 3. Effects of Other Respiratory Viruses on the Cardiovascular System

### 3.1. SARS-CoV-1

SARS-CoV-1 caused the SARS outbreaks from 2002 to 2004. Over 8000 people from more than 30 countries were infected with this virus and over 700 died [22]. SARS-CoV-1 and SARS-CoV-2 share approximately 79.5% genomic homology [23]. Like SARS-CoV-2, SARS-CoV-1 uses ACE2 as a receptor to facilitate entry into host cells [10,24]. Like SARS-CoV-2, SARS-CoV-1 also causes acute respiratory distress syndrome (ARDS), the leading cause of death in these patients. The similarities and differences between SARS-CoV-1 and SARS-CoV-2 have been reviewed elsewhere [23,25,26].

Histological examinations of postmortem patients who died of SARS have shown systemic vasculitis including edema, localized fibrinoid necrosis, and the infiltration of monocytes, lymphocytes, and plasma cells into vessel walls in the heart, lung, liver, kidney, adrenal gland, and the stroma of striated muscles [27]. Thrombosis has been found in small veins. The degeneration and necrosis of the parenchyma cells in the heart have also been observed [27]. Hwang et al. [28] observed endothelial damage to both small and medium-sized pulmonary vessels. A postmortem analysis of a 57-year-old man showed the proliferation, swelling, and apoptosis of endothelial cells and edema, inflammatory cell infiltration, and fibrinoid necrosis in the walls of small blood vessels in specimens from the lungs, heart, liver, kidneys, adrenal glands, brain, GI tract, and muscle tissues [29]. In addition, thrombi are evident in the veins and microcirculation of the soft tissues surrounding the lungs, spleen, pancreas, kidneys, adrenal glands, and mesenteric lymph nodes [29]. Liu et al. [30] found that SARS patients have increased levels of tissue plasminogen activator and soluble thrombomodulin, suggesting the possibility of endothelial injury.

An echocardiographic study of 46 patients showed that SARS causes subclinical diastolic impairment without systolic involvement [31]. Pulmonary SARS-CoV-1 infection also leads to myocardial SARS-CoV-1 infection associated with increased myocardial inflammation, interstitial fibrosis, and the pathological hypertrophy of cardiomyocytes [32]. In a study of 121 patients with SARS, hypotension and tachycardia were found to be common, bradycardia and cardiomegaly were less common, and cardiac arrhythmia was rare [33].

Infections with SARS-CoV-1 as well as the SARS-CoV-1 spike protein by itself without the rest of the virus have been shown to downregulate ACE2 in mice [34]. These authors later stated that “since SARS spike-protein-mediated ACE2 down-regulation appears to contribute to the severity of lung failure, these findings may explain how the SARS-CoV-1 has turned into a lethal virus” [35] and that the SARS-CoV-1 infection- and spike protein-mediated downregulation of ACE2 serves as an “explanation for a killer virus” [36]. Similarly to the finding in the lungs, respiratory infection with SARS-CoV-1 in mice as well as humans leads to decreased myocardial ACE2 expression [32]. Thus, the spike protein-mediated downregulation of ACE2 that could increase the level of Ang II likely promotes the pathogenesis of cardiovascular and pulmonary diseases.

Furthermore, Chen et al. [37] reported that the SARS-CoV-1 spike protein directly elicits cell signaling through the activation of the casein kinase II-dependent phosphorylation of ACE2. The authors suggested that this action of the spike protein may account for the development of fibrosis.

**Figure 1:**
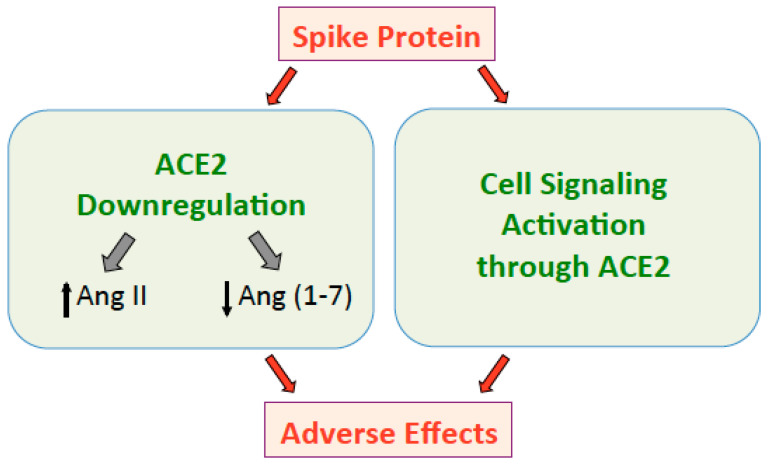
The spike protein downregulates ACE2 that in turn increases Ang II and decreases Ang (1–7), resulting in adverse effects. The spike protein also directly activates cell signaling through the ACE2 receptor, which may also promote adverse events.

Thus, it is crucial to consider the adverse actions of the spike protein to develop therapeutic agents and vaccines for COVID-19 (Figure 1). Recently, the spike protein of SARS-CoV-2 (without the rest of the virus) has also been shown to activate cell signaling in human cells [38].

### 3.2. Middle East Respiratory Syndrome-Related Coronavirus (MERS-CoV)

MERS-CoV is a coronavirus that emerged in 2012 with approximately 2500 cases [39]. This virus is highly lethal with a reported fatality rate of 34.4% [23]. MERS-CoV and SARS-CoV-2 share about 50% genomic homology [23]. MERS causes flu-like symptoms as well as severe life-threatening illnesses including ARDS, pneumonia, myocarditis, and organ failure. Like COVID-19, mortality is the highest in the elderly and individuals with pre-existing conditions such as diabetes, heart disease, hypertension, respiratory disease, renal failure, obesity, and immunodeficiency [40,41].

MERS-CoV uses dipeptidyl peptidase-4 (DPP4) expressed in type 1 and type 2 alveolar cells, the bronchial epithelium, bronchial submucosal glands, the endothelium, alveolar macrophages, and leukocytes as the receptor for spike protein-mediated membrane fusion [42]. DPP4 is an aminopeptidase that plays a critical role in glucose metabolism, and DPP4 inhibitors have been approved for the treatment of type 2 diabetes mellitus [43].

**Figure 2:**
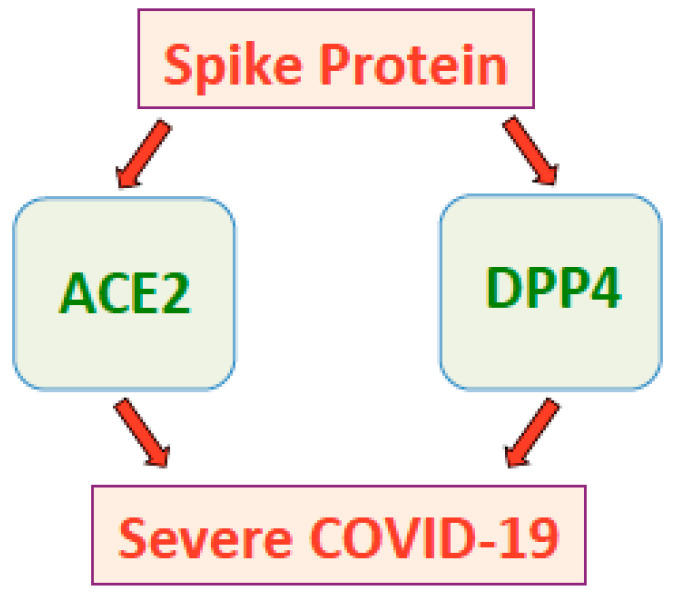
The SARS-CoV-2 spike protein promotes severe COVID-19 conditions through ACE2 and possibly DPP4.

Thus, the interaction between the SARS-CoV-2 spike protein and DPP4 could explain the link between COVID-19 and diabetes. The possible role of DPP4 in the pathogenesis of severe and fatal COVID-19 conditions that occur in certain populations of individuals should further be considered (Figure 2).

### 3.3. Influenza

The influenza virus is a negative-sense, single-stranded RNA virus that contains two surface glycoproteins. Its membrane fusion protein is hemagglutinin, which binds to host cell receptors containing sialic acid. During the release of the virus from host cells, neuraminidase cleaves terminal sialic acids [44]. Influenza commonly infects the upper respiratory tract causing a self-limiting infection. However, in severe cases, it may spread to the lower respiratory tract, causing viral pneumonia, which can progress to ARDS [45].

Severe influenza is partly mediated by the cytokine storm, particularly tumor necrosis factor-α (TNF-α) [46]. It has been shown that influenza upregulates TNF-α, interleukin (IL)-1β, and IL-6 and that these cytokines increase trypsin expression in endothelial cells. This upregulated trypsin induces the loss of zona-occludens-1 and vascular hyperpermeability [47]. Cytokines also attract leukocytes to the endothelium and these activated neutrophils produce neutrophil extracellular traps, which have been shown to have cytotoxic effects on endothelial cells and contribute to lung damage in influenza-infected mice [48]. Influenza can directly infect endothelial cells and activate NF-κB, causing upregulated cytokine and chemokine production and subsequent vascular leakage [49]. It has also been shown that agonists of S1P1, a receptor expressed in pulmonary endothelial cells, suppresses the cytokine storm and decreases mortality [50].

Thrombosis has been found in patients with influenza [51]. Influenza promotes hypoxia that induces a pro-inflammatory state in the endothelium, causing the increased release of IL-1, IL-6, platelet-activating factor, intercellular adhesion molecule (ICAM)-1, p-selectin, and von Willebrand factor, all of which are associated with platelet activation [52]. Influenza virus induces platelet adhesion to the lung endothelium, likely mediated by the interaction with endothelial fibronectin, which is thought to be thrombogenic [53].

Influenza is associated with an increased incidence of cardiovascular diseases including atherosclerosis, myocardial infarction, cardiac arrest, and stroke [54]. Myocarditis has been reported in approximately 0.4–13% of hospitalized patients with influenza [55]. Congestive heart failure is seen in 84% of patients diagnosed with myocarditis; 62% of these patients require advanced cardiac support therapies [56]. In H1N1 influenza A-infected patients, the right ventricular dysfunction is more prevalent than the left ventricular dysfunction [57]. Ludwig et al. [58] reported that 24% of veterans who tested positive for the influenza virus have acute cardiac injuries, with 49% being myocardial infarction.

In relation to COVID-19, Liu et al. [59] reported that the infection of cultured human nasopharyngeal carcinoma cell line CNE-2Z or human embryonic kidney cell line 293 T with the H1N1 influenza A virus results in the downregulation of ACE2 protein. The authors further showed that this downregulation of ACE2 is dependent on proteasomal degradation and regulated by neuraminidase. In mice, Zou et al. [60] found that H5N1 infection causes the reduction of ACE2 protein in the lungs. Similarly, mice infected with the H7N9 influenza A virus have been found to have downregulated ACE2 protein expression in the lungs [61]. Likewise, the plasma levels of Ang II are elevated in H7N9-infected patients [62].

**Figure 3:**
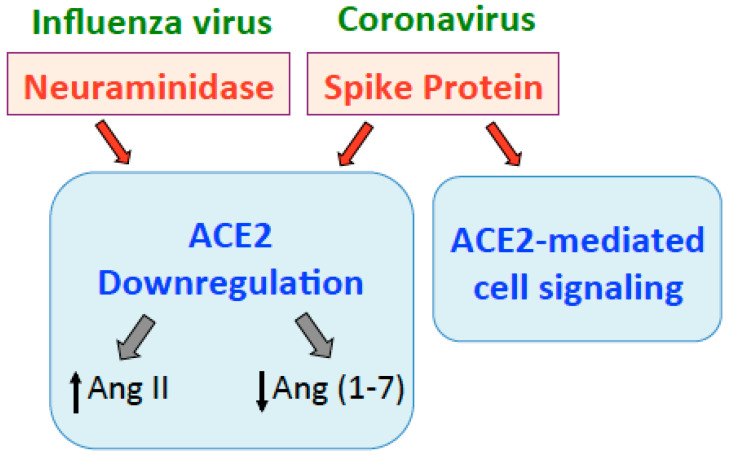
Influenza virus (through neuraminidase) and coronaviruses (through the spike protein) downregulate ACE2, increasing Ang II and decreasing Ang (1–7). The spike protein also elicits cell signaling through the ACE2 receptor.

These results suggest that the influenza virus and SARS-CoV-2 may affect the cardiovascular system in a similar manner through the downregulation of ACE2 and subsequent influence on angiotensins (Figure 3). However, the cardiovascular manifestation seems to be more severe for SARS-CoV-2 than the influenza virus. For example, while COVID-19 patients who died of ARDS due to SARS-CoV-2 infection exhibit pulmonary vascular wall thickening, patients who died of ARDS due to H1N1 influenza infection do not [38]. Thus, Ang II may not necessarily be the determinant of the viral influences on ACE2; rather, mechanism-like spike protein-mediated cell signaling (Figure 3) may play a crucial role in COVID-19 pathogenesis [38,63].

## 4. Effects of Non-Respiratory Viruses on the Cardiovascular System

### 4.1. Dengue Virus

Dengue viruses are mosquito-borne human pathogens in tropical countries. Dengue virus infections are often symptomatic or cause mild symptoms, but they can result in more severe hemorrhagic fever characterized by vascular leakage [64]. There are similarities between dengue virus and SARS-CoV-2. They are both positive-sense single-stranded RNA viruses, their clinical symptoms are similar, and both increase endothelial permeability [65].

Dengue virus infection has been associated with myocarditis, arrhythmias, left ventricular dysfunction, pulmonary edema, and tricuspid regurgitation [66]. Dengue virus also increases the risk of stroke, with the highest risk being within the first two months of diagnosis [67]. In Brazil, five cases of large vessel thrombosis have been reported to be associated with the acute phase of dengue fever [68]. Dengue virus-mediated endothelial permeability may be responsible for these cardiovascular complications.

Various factors have been shown to be involved in dengue virus-mediated endothelial permeability. In the plasma of patients with dengue hemorrhagic fever, vascular endothelial growth factor (VEGF), tryptase and chymase have been found to be significantly increased [69]. In mice, dengue virus induces widespread mast cell activation, leading to the release of chymase and leukotrienes, which activate endothelial cells, increase vascular permeability, and promote a pathological loss of vascular integrity [70]. Brown et al. [71] showed that the infection of dengue virus in mast cells results in the release of factors, which activate endothelial cells via the increased expression of ICAM-1 and VCAM-1 regulated by TNF-α. In patients infected with dengue virus, PAF, a protein involved in vascular leakage during shock and anaphylaxis, is significantly increased and serum from dengue patients downregulates ZO-1, a tight junction protein, leading to vascular leakage [72].

Children with dengue hemorrhagic fever have been found to have reduced plasma angiopoietin 1 (that maintains vascular integrity) and increased angiopoietin 2 (that promotes vascular leakage) [73]. These authors also found an inverse correlation between angiopoietin-1 and markers of plasma leakage and a positive correlation between angiopoietin-2 and plasma leakage. In patients with dengue virus infection, angiopoietin-2, endothelin-1, and matrix metalloproteinase-2 are increased and levels of soluble VEGFR-2 are decreased, and this pattern is associated with vascular leakage [74].

Further, dengue virus has been shown to induce vascular leakage in mice by activating the NLRP3 inflammasome to release IL-1β [75]. The activation of NLRP3 inflammasome has also been implicated in SARS-COV-2 infection [76].

High levels of dengue virus non-structural protein 1 (NS1), a secreted glycoprotein involved in viral replication and immune evasion, have been correlated with disease severity and the development of dengue hemorrhagic fever [77]. In vivo and in vitro studies have shown that NS1 alone (without the rest of the viral components) can increase endothelial barrier permeability and cause vascular leakage, which can be attenuated with an NS1 vaccine [78]. Cheung et al. [79] also reported that NS1 specifically disrupts the ability of pericytes to support endothelial cell function in a three-dimensional microvascular assay, leading to endothelial hyperpermeability. In human endothelial cells, NS1 activates p38 mitogen-activated protein kinase and the inhibitor of this kinase reduces NS1-mediated endothelial permeability [80].

The studies by Beatty et al. [78] used the dengue virus NS1 protein at 10 mg/kg body weight for mouse experiments to elicit endothelial permeability, while the studies by Kuba et al. [34] used 1 mg/kg body weight of the SARS-CoV-1 spike protein to elicit the Ang II-dependent augmentation of lung injury. In cell culture studies, approximately 20 nM [78], 10 nM [79], and 200 nM [80] concentrations of dengue virus NS1 protein were used to elicit biological responses. Conversely, approximately 0.1 nM of the SARS-CoV-2 spike protein elicits biological responses [38]. These in vivo and in vitro experiments may indicate that coronavirus spike proteins are more effective in eliciting biological effects on host cells than dengue NS-1.

### 4.2. Human Immunodeficiency Virus (HIV)

HIV is a retrovirus that uses reverse transcriptase to synthesize DNA from its RNA genome to integrate into the host cell genome. HIV uses its membrane fusion protein gp120 to bind to the cluster of differentiation (CD4) protein on the surface of immune cells such as T helper cells, monocytes, macrophages, and dendritic cells [81].

HIV infection has been associated with a wide variety of cardiovascular diseases. In one cohort of 840 HIV patients, 17 were found to have a coronary event with acute coronary syndrome being the first manifestation in 14 patients [82]. The prevalence of peripheral artery disease is high in the HIV-infected population and occurs around 20 years earlier than in the general population [83]. Low CD4 counts are an independent risk factor for cardiovascular diseases [84]. HIV patients, particularly women, have been found to have increased rates of acute myocardial infarction than the general population [85]. Post et al. [86] reported that HIV-infected men have an increased prevalence of coronary artery plaques, especially non-calcified plaques, compared with uninfected men independent of other coronary artery disease risk factors. Pericardial effusion is the most common cardiovascular complication of HIV, with an incidence of 11–17% per year [87]. HIV has been implicated as a cause of myocarditis, the mechanism of which is unknown. Anti-alpha myosin autoantibody concentrations are greater in HIV patients than controls, suggesting that cardiac autoantibodies may be markers of the development of left ventricular dysfunction in patients with HIV [88]. Left ventricular dilation or hypokinesis has been found in 23% of HIV-infected patients [89]. Currie et al. [90] showed a correlation between dilated cardiomyopathy and a poor prognosis compared with HIV-infected patients with normal hearts. In the postmortem analysis of eight HIV-seropositive patients, the distal and proximal vascular lesions of coronary arteries were observed as exhibited by the presence of atherosclerosis, fibrosis, and sclero-hyalinosis [91]. HIV can also cause vasculitis with histopathologic findings including necrotizing arteritis, nonnecrotizing arteritis, neutrophilic inflammatory vascular disease, mononuclear inflammatory vascular disease, and other small-vessel inflammatory changes [92]. HIV patients are also at a higher risk of developing hypertension at a younger age than the general population [93]. Furthermore, HIV is an independent risk factor for the development of pulmonary arterial hypertension (PAH). The estimated overall incidence of PAH in HIV patients is 0.5% [94,95,96,97]. PAH is considered to be an independent predictor of mortality among HIV-positive patients [98]. The levels of endothelin-1 are associated with the incidence of PAH in the HIV-infected population [99].

HIV gp120, a glycoprotein that initiates viral entry into cells by interacting with its host cell receptor, CD4, and co-receptors, CXCR4 and CCR5, is partially responsible for HIV-associated cardiovascular complications. It has been shown that gp120 binds to CXCR4 in endothelial cells and causes apoptosis [100]. These apoptotic effects have been shown to be mediated through caspases [101], protein kinase C [102], and p38 mitogen-activated protein kinase [103]. In addition, gp120 has been shown to induce the production of reactive oxygen species, which could contribute to endothelial dysregulation [104]. The treatment of human brain endothelial cells with gp120 increases endothelial permeability and decreases tight junction proteins Zo-1, ZO-2, and occluding [105]. This could explain how the virus enters the nervous system, thereby causing neurological diseases associated with HIV. Furthermore, gp120 upregulates IL-6 and IL-8 through signal transducer and activator of transcription 1 (STAT1) signaling, leading to the margination and migration of monocytes through the blood–brain barrier [106]. In a study conducted on human lung microvascular endothelial cells, it was shown that gp120 increases the expression of endothelin-1 and could lead to PAH in patients with HIV [107]. In human pulmonary artery smooth muscle cells, gp120 activates MEK [108], increases intracellular calcium and induces cell growth [109]. gp120 also activates protein kinase C and reactive oxygen species signaling in human vascular smooth muscle cells [110].

HIV Tat is a regulatory protein that enhances viral transcription. Tat downregulates tight junction proteins—namely, claudin-1, claudin-5, and ZO-2—leading to increased endothelial cell permeability [111]. Tat protein has also been shown to be an angiogenic factor. Albini et al. [112] showed that Tat binds to Flk-1/KDR, a VEGF-A tyrosine kinase receptor in endothelial cells, causing angiogenesis. Tat can also cause the apoptosis of endothelial cells through caspase activation [113]. Tat and TNF-α work synergistically to induce endothelial activation and promote damage [114].

HIV Nef has been shown to induce apoptosis by the generation of reactive oxygen species and increase MCP-1, an atherogenic protein, in endothelial cells [115]. Nef can also induce angiogenesis while acting synergistically with Kaposi’s sarcoma-associated herpesvirus oncoprotein K1 [116].

**Figure 4:**
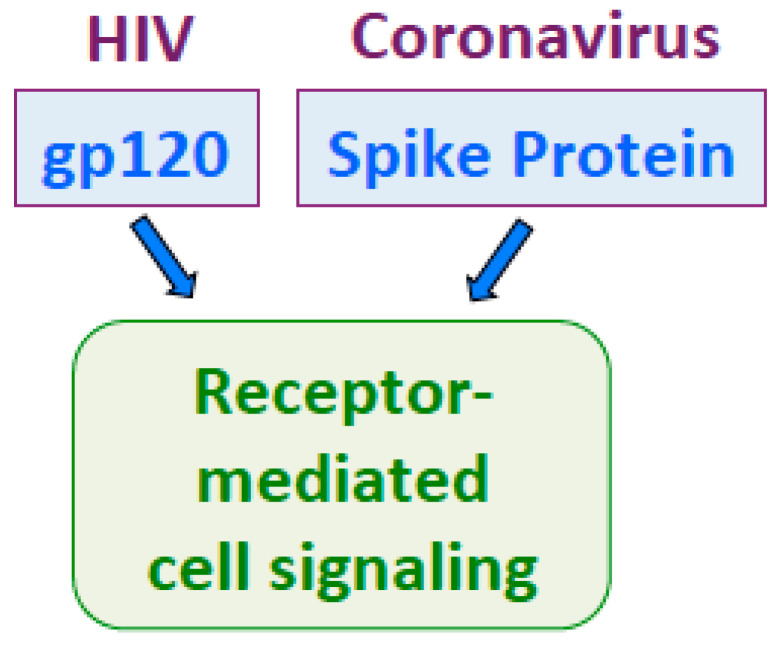
HIV gp120 and the spike protein both activate receptor-mediated cell signaling at picomolar concentrations.

Like SARS-CoV-2 spike protein [38], HIV gp120 activates cell signaling at low concentrations around 100 pM [108,109]. Thus, these proteins may exhibit similar properties as human growth factors. Understanding cell signaling mechanisms by the spike protein and gp120 may be critical to overcome these deadly viruses (Figure 4).

## 5. Discussion

The present work discussed the effects of various viruses on the cardiovascular system/diseases with a focus on molecular mechanisms to provide information that may be useful for developing preventive and treatment strategies for SARS-CoV-2 infection and COVID-19. The literature searches performed indicated the importance of ACE2 downregulation and increased Ang II levels by SARS-CoV-2, SARS-CoV-1 and the influenza virus in the occurrence of cardiovascular complications in patients infected with these viruses. The spike protein appears to be responsible for the downregulation of ACE2 by these coronaviruses, while influenza-mediated ACE2 reduction occurs through neuraminidase. In addition, the SARS-CoV-2 and SARS-CoV-1 spike proteins directly elicit cell signaling through the activation of ACE2 by converting this protease to a membrane cell signaling receptor, potentially leading to cardiovascular disorders. DPP4 activation by the spike protein as seen in MERS-CoV may also be important in COVID-19, especially considering the role of diabetes. Dengue virus has been found to release NS-1, which may promote cardiovascular complications; however, its effects may not be as potent as the actions of the spike proteins of SARS-CoV-1 and SARS-CoV-2. Strong evidence based on a number of studies points to the role of HIV gp120 in cardiovascular complications in patients infected with this virus. Similarities between gp120 and the spike protein should further be explored to determine the possible mechanisms shared by the two viruses (i.e., SARS-CoV-2 and HIV) that have been the major sources of the health crisis. The actions of these viral membrane fusion proteins on the cardiovascular system should carefully be considered to develop safe and effective vaccines [108,117].
